# Treatment resistant FEP (first episode of psycosis) with neuroanatomical findings

**DOI:** 10.1192/j.eurpsy.2024.1514

**Published:** 2024-08-27

**Authors:** K. Papageorgiou, I. Retsou, E. Papadopoulou, D. Antoniadis

**Affiliations:** Psychiatric Hospital of Thessaloniki, Thessaloniki, Greece

## Abstract

**Introduction:**

Presentantion of the first psychotic episode of a young man and the investigation of the efficacy of treatment with olanzapine and after cariprazine.

**Objectives:**

Assessing the response to treatment of cariprazine in a psychotic patient with relevant neuroanatomical findings.

**Methods:**

A 25-year old man was admitted to the psychiatric intensive care due to his agggressive behavior and verbal abuse, threatening to kill them both. His medical history included long periods of negatively affected mood, social isolation and talking to himself according to his family

**Results:**

Whent the patient was admitted he was very anxious, alert and extremely aggressive. During the interview he admitted auditory and visual hallucinations alongside delusional ideation with a particular aggression towards his father.

Upon admission his PANSS score was 121. positive scale score was 23.

The patient was treated initially with monotherapy olanzapine, gradually increased up to 20mg OD. Olanzapine caused asymptomatic transaminasemia, a relatively common adverse effect. At this point a change in medication was made and olanzapine was stopped and cariprazine was added gradually increasing its dose from 1,5mg to 6mg OD.

Interestingly the medical investigations (brain CT scan) indicated a calcification in falx cerebri.

After a period of 48 days since admission the patient was clinically improved and was discharged. His PANSS score was 72. Positive scale was 10.

**Conclusions:**

The use of cariprazine as a treatment for a first psychotic episode of a young male improved his PANSS score after a 22-day treatment. According to the literature neuroanatomical findings have been associated with poor prognosis regarding the course of the illness. There needs to be further investigation on the efficacy of the long term treatment for this patient.

**Disclosure of Interest:**

None Declared

